# Residents' views about family medicine specialty education in Turkey

**DOI:** 10.1186/1472-6920-10-29

**Published:** 2010-04-15

**Authors:** Arzu Uzuner, Pinar Topsever, Ilhami Unluoglu, Ayse Caylan, Nezih Dagdeviren, Yesim Uncu, Mumtaz Mazıcıoğlu, Alis Ozçakır, Hakan Ozdemir, Fusun Ersoy

**Affiliations:** 1 Family Medicine Department, Marmara University Medical School, Altunizade, Istanbul, Turkey; 2 Family Medicine Department, Acıbadem University Medical School, Kozyatağı, Istanbul, Turkey; 3 Family Medicine Department, Eskişehir Osmangazi University Medical School, Eskişehir, Turkey; 4 Family Medicine Department, Trakya University Medical School, Edirne, Turkey; 5 Family Medicine Department, Uludağ University Medical School, Bursa, Turkey; 6 Family Medicine Department, Erciyes University Medical School, Kayseri, Turkey; 7 Ministry of Health, General Directorate of Primary Health Care Systems, Family Medicine Department Ankara, Turkey

## Abstract

**Background:**

Residents are one of the key stakeholders of specialty training. The Turkish Board of Family Medicine wanted to pursue a realistic and structured approach in the design of the specialty training programme. This approach required the development of a needs-based core curriculum built on evidence obtained from residents about their needs for specialty training and their needs in the current infrastructure. The aim of this study was to obtain evidence on residents' opinions and views about Family Medicine specialty training.

**Methods:**

This is a descriptive, cross-sectional study. The board prepared a questionnaire to investigate residents' views about some aspects of the education programme such as duration and content, to assess the residents' learning needs as well as their need for a training infrastructure. The questionnaire was distributed to the Family Medicine Departments (n = 27) and to the coordinators of Family Medicine residency programmes in state hospitals (n = 11) by e-mail and by personal contact.

**Results:**

A total of 191 questionnaires were returned. The female/male ratio was 58.6%/41.4%. Nine state hospitals and 10 university departments participated in the study. The response rate was 29%. Forty-five percent of the participants proposed over three years for the residency duration with either extensions of the standard rotation periods in pediatrics and internal medicine or reductions in general surgery. Residents expressed the need for extra rotations (dermatology 61.8%; otolaryngology 58.6%; radiology 52.4%). Fifty-nine percent of the residents deemed a rotation in a private primary care centre necessary, 62.8% in a state primary care centre with a proposed median duration of three months. Forty-seven percent of the participants advocated subspecialties for Family Medicine, especially geriatrics. The residents were open to new educational methods such as debates, training with models, workshops and e-learning. Participation in courses and congresses was considered necessary. The presence of a department office and the clinical competency of the educators were more favored by state residents.

**Conclusions:**

This study gave the Board the chance to determine the needs of the residents that had not been taken into consideration sufficiently before. The length and the content of the programme will be revised according to the needs of the residents.

## Background

In Turkey, specialty training in Family Medicine has been offered in Education and Research State Hospitals since 1985 (allied with the Ministry of Health, MoH) and in Academic Family Medicine Departments allied with medical schools since 1993 [1,2]. Family Medicine has been included since 1983 as a specialty in the Standing Rules of the Medical Specialties (Tıpta Uzmanlık Tüzüğü, TUT) of the MoH which defines the overall structure, standards and implementation of residency training for all medical specialties [3].

According to the TUT, Family Medicinespecialty programmes have to feature the following external block rotations:

• 9 months of internal medicine

• 9 months of pediatrics

• 8 months of obstetrics and gynecology (Obs&Gyn)

• 6 months of emergency medicine (predominantly surgery)

• 4 months of psychiatry

with a total duration of 36 months (3 years) [2,3].

In 2005 Family Medicine residency training was provided in the inpatient and outpatient clinics of 11 education and research state hospitals and in 27 university Family Medicine Departments [4,5]. In 2005 Family Medicine residency programmes at the state hospital level under the auspices of the MoH were coordinated by the department heads of other clinical disciplines named "clinic chiefs", whereas in university departments postgraduate education was managed by academic family physicians. Although university-based academic departments of Family Medicine are more numerous compared to state hospitals (27 vs 11), the majority of Family Medicine residents in Turkey are trained in education and research state hospitals. According to data from the Ministry of Health in 2005, only 36 of a total of 511 residency positions were offered by university Family Medicine Departments (7.04%). University departments usually adopt the basic schedule as indicated in the TUT for the Family Medicine residency programme, but to improve the quality of the residency programme, academic departments usually provide modifications that result in an extension of the duration of the programme. In Turkey, the development of a core curriculum for residency programmes was parallel to the developments in the world. The boards of specialty associations play a key role in this movement. Core curriculum and residents' carnet are important tools needed for standardization, objectivity and the assessment of postgraduate education. Apart from being a sine qua non for quality assurance, the standardization of training is a preliminary condition for international accreditation of professional qualifications [6]. All postgraduate education programmes are being revised in this context [7,8].

Family Medicine is a specialization of primary care, and the content of the residency training should be defined according to the needs of the care [9].

The Turkish Board of Family Medicine (TAHYK), founded in 1998, held its first elections for executive board membership in 2003, and planned preliminary activities to improve residents' training. In this context, the first task of the committee has been to prepare the core curriculum and the resident assessment form and to revise the ongoing residency training programme with a health-care-needs, competency and evidence-based approach. With this aim, Family Medicine specialty programmes from different countries and Family Medicine textbooks have been analyzed. A workshop has been organized to determine the expectations of family physicians who have been involved in Family Medicine residency training as trainers as well as trainees. To design a contemporary curriculum of high quality, it is important to collect the views of all stakeholders [10]. Thus, the TAHYK planned a survey to collect residents' views to make an assessment of needs.

This study was designed with the aim of revealing Family Medicine residents' views about their specialty training.

## Methods

This is a descriptive, cross-sectional study. The board (TAHYK) executive committee prepared a questionnaire to investigate residents' views about characteristics of the education programme (such as duration and content), to assess residents' learning needs as well as needs for a training infrastructure. The questionnaire included five essential topics frequently discussed: 1) duration of the specialty training and of the internal and external rotations, 2) arrangement of the rotations, the need for new ones and a needs assessment for subspecialties, 3) assessment of current training methods and the need for other/new methods, 4) expectations of the physical conditions of a training unit and 5) quality criteria for the trainers. In addition to closed-ended questions, open-ended questions allowed the participants to indicate precise suggestions about the timing and types of rotations. Furthermore, a three-item Likert scale (necessary, preferential, no need) helped to define views about the training methods, physical conditions and quality of the trainers.

The questionnaire was sent to all of the Family Medicine Departments in the universities (n = 27) and to the coordinators in State Education and Research Hospitals (n = 11) by mail, e-mail and personal contact.

A total of 191 questionnaires were returned from nine state hospitals and ten university departments of Family Medicine. In 2005 a total of 664 Family Medicine residents (511 new entrants plus 153 continuing vocational trainees) were estimated to be in training countrywide [11].

Despite significant efforts, only a low percentage (28.8%) of the residents could be reached. Qualitative methods could have been more suitable for reflecting the views of the residents. These can both be cited as limitations of the study.

The statistical analysis of the study was performed by using the SPSS 11.0 statistics programme. The frequencies for the choices and mean values for the duration of the rotations have been calculated. Non-parametric tests were used to compare university and state residents' responses.

## Results

### Participants and response rates

The university/state hospital participant ratio was 35.1% (n = 67)/64.9% (n = 124) from a total of 19 institutions. The female/male ratio was 58.6% (n = 112)/41.4% (n = 79). The main items of the survey with response rates are summarized in Table 1.

**Table 1:** Response rate to questions of the survey

	Yes		No		Total	
	
Questions	%	N	%	n	%	n
Is the duration of residency training sufficient?	55.0	104	45.0	85	100	189

Is the duration of the rotations adequate?	42.6	80	57.4	108	100	188

Should rotations in other departments be included?	88.8	166	11.2	21	100	177

Should family medicine have sub-specialties?	47.4	82	52.6	91	100	173

Should a rotation at a private primary care center be included?	59.4	111	40.6	76	100	187

Should a rotation at a state primary care center be included?	62.8	115	37.2	68	100	183

### The duration of the residency programme

Forty-five percent of the respondents were not satisfied with the duration of the residency programme (n = 85). University residents (n = 33) were more likely to approve of the present rotation periods than MoH residents (n = 47) (50.8% university vs 38.2% MoH, *p *= 0.067). Fifty-eight percent of the unsatisfied participants thought that 4 years could be better than 3 years; 28% suggested for a duration of 5 years, 5% for 4.5 years, 4% for 6 years, 3% for 3.5 years, 1% for 1 year and 1% for 8 years.

### Residents' thoughts about the optimal programme length and the length of the rotations

#### Different lengths for the standard rotations have been proposed (Figure 1)

**Figure 1:**
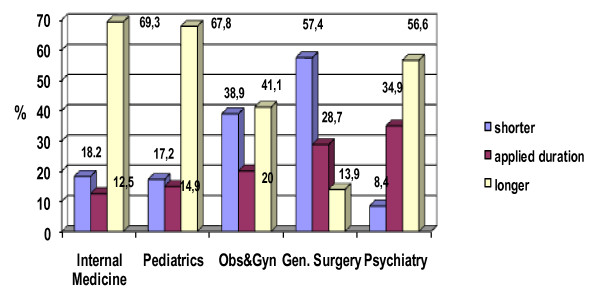
**Preference for standard rotation periods by proportion of respondents**.

The length of the internal medicine rotation is nine months; 12.5% (n = 11) of the residents accepted the nine -month duration, 56.9% (n = 50) proposed 10-12 months, 15.9% (n = 14) proposed one to six months, 12.5% (n = 11) proposed more than 12 months and 3.4% (n = 3) proposed seven to eight months.

The duration of the rotation in pediatrics is nine months; 14.9% (n = 13) of the residents agreed with eight months, 56.3% (n = 49) of the participants proposed 10-12 months, 17.2% (n = 15) proposed one to eight months and 11.4% (n = 10) proposed more than 12 months.

The duration of the rotation in obstetrics and gynecology is eight months; 20.0% (n = 18) of the participants agreed with eight months, 23.3% (n = 21) of the participants proposed 9-10 months, 20.0% (n = 18) proposed six months, 17.8% (n = 16) proposed 12 months and over, 16.6% (n = 15) proposed one to five months and 2.2% (n = 2) proposed seven months.

The general surgery rotation is six months; 28.7% (n = 29) of the participants agreed with six months, 57.4% (n = 58) proposed less than six months and 13.9% (n = 14) proposed more than six months.

The current duration of the psychiatry rotation is four months; 34.9% (n = 29) agreed with four months, 39.8% (n = 33) of the participants proposed six months, 16.8% (n = 14) proposed eight months or longer and 8.4% (n = 7) proposed three months.

### The need for other rotations

A total of 88.8% of the participating residents suggested a need for rotations beyond the standard programme. Residents from state hospitals (n = 112) expressed the need for other rotations more frequently than university residents (n = 54) (93.3% MoH vs. 80.6% university, *p *= 0.008, OR = 1.2, 95% CI = 1.02-1.32). The proposed rotations are summarized in Table 2.

**Table 2:** Other rotations proposed by the participants and current practice

	Proposed			Current practice		
**Rotations**	**Duration(wk)mean ± SD**	**N**	**%**	**Duration(wk)** **mean ± SD**	**n**	**%**

Dermatology	5.4 ± 3.6	118	61.8	3.8 ± 0.7	31	16.2

Otolaryngology	5.6 ± 4.5	112	58.6	3.5 ± 1.2	33	17.3

Radiology	5.7 ± 4.3	100	52.4	2.5 ± 0.8	6	3.1

Ophthalmology	4.7 ± 3.9	74	38.7	2.8 ± 1.2	4	2.9

Cardiology	6.5 ± 3.5	71	37.2	3.9 ± 0.5	37	19.4

Neurology	4.9 ± 3.8	64	33.5	3.0 ± 1.4	2	1.0

Geriatrics	4.9 ± 3.7	59	30.9	8.0 ± 0.0	1	0.5

Pediatric psychiatry	5.8 ± 3.5	57	29.8	4.0 ± 0.0	21	10.9

Pediatric pulmonology	5.2 ± 4.4	40	20.9	3.5 ± 1.0	4	2.1

Pediatric cardiology	4.5 ± 2.4	37	19.4	2.1 ± 0.5	19	9.9

Gastroenterology	4.9 ± 3.6	32	16.8	4.3 ± 2.1	16	8.4

Pediatric allergy	3.7 ± 2.3	30	15.7	0.0 ± 0.0	0	0.0

Rheumatology	4.4 ± 3.2	29	15.2	3.7 ± 1.1	13	6.8

Nephrology	4.6 ± 2.5	25	13.1	3.6 ± 1.1	15	7.9

Pediatric neurology	4.2 ± 3.2	24	12.6	0.0 ± 0.0	0	0.0

### Practice in a Primary Care Centre (PCC)

In Turkey, primary care is provided in the private and public sectors. MoH primary care centres (MoH health centres) are spread all over the country according to population size; private health centres are the private offices of family physicians and general practitioners. The residents believe in the need for a rotation in a primary healthcare centre; 59.4% (n = 111) of the respondents proposed a rotation in a private centre, 62.8% (n = 115) in a MoH health centre and 46.1% (n = 88) of the participants indicated the need for field rotations in private as well as MoH health centres. University residents (n = 50) were much more likely to generally approve of rotations in PCCs than MoH residents (n = 61) (79.4% university vs. 49.2% MoH, *p *< 0.001 OR = 4, 95% CI = 2-8). The length of training in a private primary care centre is three months (median) with a range of 0.5-24 months and three months (median) with a range of 0.5 to 18 months for a MoH health centre. See Table 3.

**Table 3:** Duration of primary care practice proposed by the residents

	Primary care			
	Private (N = 108)		State (N = 108)	
	
Duration (month)	n	%	n	%
<6 mo	70	64.8	62	57.4

≥ 6	38	35.2	46	42.6

* ≥ 12 months: 9.2%, n = 10 for public PCC and 12.0%, n = 13 for private PCC

### Proposed subspecialties

A total of 47.4% (n = 82) of the participating residents stated the necessity for adding subspecialties; university (n = 35) and MoH residents (n = 51) did not differ with respect to their preference in terms of views on the necessity of additional subspecialties (45.5% MoH residents vs 50.8% university, *p *= 0.31). Geriatrics was the most frequently proposed subspecialty, mentioned by 16.3% (n = 32) of the participants. Less frequently cited other subspecialties were family counseling, home care, occupational medicine, family planning, mother and child care, family therapy, anti-aging, algology, social psychiatry, sports medicine, endocrinology and pediatric psychiatry. In addition to these special interest areas, some main specialties such as pediatrics, internal medicine, psychiatry and obstetrics were cited.

### Educational methods

The most commonly used educational methods in the state hospitals and in the Family Medicine Departments were residents' seminars (82.4%, n = 60), lectures given by educators (59.4%, n = 44) and case presentations (41.3%, n = 31). The views of the residents about the necessity of the educational methods are shown in Table 4.

**Table 4:** Residents preference of educational methods and their current use

	Necessity							
		
	Necessary		Preferential		No need		Current use n = 118	
	
Methods	N	%	n	%	n	%	n	%
Seminaries presented by residents n = 151	105	69.5	43	28.5	3	2.0	**60**	**50.8**

Lessons given by the educators n = 156	88	56.4	65	41.7	3	1.9	**44**	**37.3**

Case presentations n = 171	98	57.3	71	41.5	2	1.2	**31**	**26.3**

Problem based learning n = 150	79	52.7	70	46.7	1	0.7	**29**	**24.6**

Coaching n = 104	54	51.9	28	26.9	21	20.2	**24**	**20.3**

Debate n = 140	89	63.6	38	27.1	13	9.3	**5**	**4.2**

Training with models n = 151	78	51.7	38	25.1	35	23.2	**2**	**1.7**

Workshops n = 103	53	51.5	23	22.3	27	26.2	**1**	**0.8**

e-learning n = 145	84	57.9	57	39.3	4	2.8	**0**	**0.0**

Workshops were more frequently preferred by university residents (n = 30) than MoH residents (n = 46) (90.9% university vs 65.7% MoH, *p *= 0.005, OR = 5.2, 95% CI = 1.4-18.9). There was no difference between university and the MoH residents with respect to their preferences about other educational methods.

Other educational facilities such as courses, congresses and affiliation (collaboration with another institution for a well defined learning need) were considered necessary (47.1%, n = 90; 63.9%, n = 122; 60.7%, n = 116, respectively) and preferred (45.0%, n = 86; 27.7%, n = 53; 28.3%, n = 54, respectively).

### Physical infrastructure

The minimum requirements for a Family Medicine training centre (clinic/department), such as a department office, a private room for the residents, a meeting room, outpatient clinics or a practice centre, a library, computer(s) and the Internet were listed, and the residents were asked to mark the necessity of the various elements of physical infrastructure. Residents' responses are summarized in Table 5.

**Table 5:** The necessity of physical conditions according to the residents' views

	Necessity					
	
	Necessary		Preferential		No need	
	
Physical conditions	n	%	N	%	n	%
Department office n = 175	69	39.4	104	59.4	2	1.1

Resident office n = 179	86	48.0	93	52.0	0	0.0

Meeting room n = 173	96	55.5	76	43.9	1	0.6

FM outpatient clinics n = 177	66	37.3	109	61.6	2	1.1

Practice centre n = 177	69	39.0	107	60.5	1	0.6

In patient clinic (service) n = 153	67	43.8	62	40.5	24	15.7

Library n = 175	97	55.4	76	43.4	2	1.1

Computer n = 171	69	40.4	101	59.1	1	0.6

Internet n = 173	72	41.6	101	58.4	0	0.0

The presence of a department office in the sense of a room/rooms for the use of the members of the department of Family Medicine was more significant for MoH residents (n = 76) than for university residents (n = 28) (67.3% MoH vs. 46.7% university, *p *= 0.008, OR = 1.4, 95% CI = 1.1-1.9); computer availability was more important for university residents (n = 43) than for MoH residents (n = 58) (69.4% university vs. 53.7% MoH, *p *= 0.046, OR = 1.9, 95% CI = 1.01-3.8).

#### Trainers

The characteristics that should be present in Family Medicine trainers according to the residents' views are shown in Table 6.

**Table 6:** Characteristics of the trainers according to the residents

	Necessity					
	
	Necessary		Preferential		No need	
	
Characteristics	n	%	N	%	N	%
Researcher identity n = 178	88	49.4	88	49.4	2	1.1

Clinical sufficiency n = 182	67	36.8	115	63.2	0	0.0

Communication skills n = 156	80	51.3	75	48.1	1	0.6

Attainability n = 173	89	51.4	82	47.4	2	1.1

Educator identity n = 180	70	38.9	108	60.0	2	1.1

MoH residents (n = 83) were more likely to rate clinical competency "preferential" than university residents (n = 32) (69.2%, n = 83 MoH vs 51.6% university, *p *= 0.02, OR = 1.34, 95% CI = 1.02-1.75).

## Discussion

This study reflects the views of nearly 30% of the Turkish Family Medicine residents in 2005. Most of the participating residents were from Ministry of Health hospitals. The length of the programme and the rotations had always been a topic of discussion among decision makers, trainers and residents. The duration of the residency was appropriate according to 55% of the participants. Although residents trained in university departments seemed to agree more often with the length of the training, this difference was not statistically significant. This might be explained by the fact that, in Turkey, Family Medicine residency programmes in university departments are usually extended to four years [5] instead of the standard 36 months according to the standing rules in the TUT [3]. Fifty-eight percent of the unsatisfied participants proposed four years as a suitable period. The length of the programme in other countries varies between three and five years. The UEMO (European Union of General Practitioners) consensus document of 1994 on 'specific training for general practice' argued the need to prolong the period of training to a minimum duration of three years [12,13].

In a similar survey performed in the United States, 65.2% of the 442 participating 3rd-year residents stated that the optimal Family Medicine residency programme length was three years; however, 37.1% favored a change to a 4-year residency [14]. "The broad scope of family medicine" was the main justification for extending residency training. Financial causes were the main reason for the reluctance for a 4th year of training since the salaries of the residents were low. The reasons for the dissatisfaction of Turkish Family Medicine residents might be low salaries and mandatory service after the completion of specialty training.

Even though the valid criteria should be "the time needed to train a family physician according to the implications of its definition and the content of the training", in fact the needs for family physicians workforce in the country is the real determinant of administrative incentives with respect to the length of the residency training [9,12].

The residents proposed different durations for the standard rotations.

The majority of the participants (70%) proposed an extension of their current rotation times in internal medicine and pediatrics from nine months to over 10 months. Similarly, it was proposed by the majority of the respondents that the psychiatry rotation of four months be extended. Six months for the general surgery rotation was too long for 57.4% of the residents. To our knowledge, no study has investigated the issue of the duration of the rotations in the Family Medicine residency programme from the perspective of the residents.

The majority (88.8%) of the residents expressed the need for rotations other than the standard ones. Family Medicine is one of the newer specialty areas in Turkey, and the residency programme formerly provided by the MoH has not been modified since 1983. The standard training programme fails to respond to the changing job definition. Residents' individual learning needs should be taken into consideration. The results of this study showed that there was a need for extra rotations such as dermatology, otolaryngology, radiology, ophthalmology, and cardiology. Residents from MoH hospitals more often expressed the need for extra rotations compared to university residents. In the study of Duane et al, the areas for which the residents needed more support were office procedures, practice management, children's skin conditions, sports medicine and emergency medicine [14].

The residency training at universities provides opportunities to modify the standard programme according to learning needs, facilitated by the rich infrastructure of university hospitals (e.g., presence of subspecialties) and academic internal dynamics that enable Family Medicinedepartments to plan and coordinate those modifications.

The UEMO consensus document-94 stated that a minimum of 50% of clinical training time should be spent in a general practice environment [12,13]. The questions about primary care rotations were answered affirmatively by more than half of the residents, which indicates approval of the need for practice-oriented training in a community-based PCC. The appropriate duration of training in a PCC was perceived to be six months or less by most of the respondents. University residents were more likely to advocate training in a primary care centre. This might be explained by the fact that academic Family Medicine departments proactively make their residents face the principles of the discipline, and thus, university residents are more likely to realize the necessity of a rotation in primary care. A rotation in primary care, which is not yet standard in our country, should be implemented in the standard programme.

Nearly half of the residents supported the implementation of subspecialties. The most frequently mentioned area was "geriatrics." The ageing of the population is an issue that constitutes a future challenge for healthcare in Turkey. The need for training Family Medicine residents to care for older patients and the chronically ill should be mirrored in the specialty training programme. This trend is in accordance with experiences in the U.S. [15].

The most commonly used educational methods in state hospitals and universities are seminars, lectures and case presentations. This is similar to the training modalities of many other specialty programmes in Turkey [3]. Problem-based learning and coaching are used less frequently, but they were rated as necessary and preferential by 99.4% and 78.8% of the residents, respectively. Even though not frequently practiced, the necessity for debate, training with models and workshops was expressed by 90.7%, 76.8% and 73.8% of the residents. Courses, congresses and affiliations were also considered necessary and "preferential", needed by 90% of the participants. Economic barriers prevent residents from participating in congresses. Affiliation is a type of collaboration for facilitating access to clinical or educational opportunities in another healthcare setting that cannot be provided in the resident's training centre. Affiliation, however, cannot be implemented without appropriate regulations by the MoH [16].

As for the physical infrastructure that should be present in a Family Medicine unit, nearly all of the respondents approved all suggestions in the questionnaire. The need for a department was more frequently expressed by MoH residents. This result could be due to the lack of a Family Medicine unit/department in state hospitals, which means no room to change their clothes, to study, to meet colleagues, to share daily problems and no meeting room for educational activities. As the university departments have their own space in the medical faculties, the residents do not feel they are different from the residents in other disciplines.

## Conclusions

Residents are one of the stakeholders of specialty training. This study gave us the chance to determine the needs of the residents that has not been taken into consideration sufficiently before. The length and the content of the programme will be revised according to the needs of the residents, and according to the projections of the country's needs. Further studies will be needed to assess the programme after the implementation of a core curriculum.

## Competing interests

The authors declare that they have no competing interests.

## Authors' contributions

AU has made substantial contributions to conception and design, acquisition of data, drafting the manuscript and revising it critically. PT has made substantial contributions to conception and design, analysis and interpretation of data, drafting the manuscript and revising it critically. IU has made substantial contributions to conception and design and has been involved in drafting the manuscript and in revising critically for important intellectual content. AC has participated in the collection of data and coordination of the study. ND has participated in the data collection and in revising the manuscript critically.

YU has been involved in the conception and design of the study and in revising the manuscript for the correct use of English. MM participated in the collection and the analysis of data. AO participated in the collection and the interpretation of data. HO has been involved in the design of the study and in drafting the manuscript. FE participated in the design of the study and participated in revising for language.

All authors read and approved the final manuscript.

## Pre-publication history

The pre-publication history for this paper can be accessed here:

http://www.biomedcentral.com/1472-6920/10/29/prepub
